# The Current Trend of Radiation Therapy for Patients with Localized Prostate Cancer

**DOI:** 10.3390/curroncol30090587

**Published:** 2023-09-01

**Authors:** Kazuyuki Numakura, Mizuki Kobayashi, Yumina Muto, Hiromi Sato, Yuya Sekine, Ryuta Sobu, Yu Aoyama, Yoshiko Takahashi, Syuhei Okada, Hajime Sasagawa, Shintaro Narita, Satoshi Kumagai, Yuki Wada, Naoko Mori, Tomonori Habuchi

**Affiliations:** 1 Department of Urology, Akita University Graduate School of Medicine, Akita 010-8543, Japan; qqc83rkd@piano.ocn.ne.jp (M.K.); yumina.muto.0601@gmail.com (Y.M.); hiromisato2002@yahoo.co.jp (H.S.); backup.sekine.bs@gmail.com (Y.S.); sobusan.sobusan.com@gmail.com (R.S.); joseph.aoyama@gmail.com (Y.A.); yopico.t@gmail.com (Y.T.); sh.ok.gn10.phy2@gmail.com (S.O.); sasahazi.820@gmail.com (H.S.); naritashintaro@gmail.com (S.N.); thabuchi@gmail.com (T.H.); 2 Department of Radiology, Akita University Graduate School of Medicine, Akita 010-8543, Japan; skuma@med.akita-u.ac.jp (S.K.); ywada@med.akita-u.ac.jp (Y.W.); nmori@med.akita-u.ac.jp (N.M.)

**Keywords:** prostate cancer, radiation therapy, intensity-modulated radiation therapy, image-guided radiation therapy, volumetric modulated arc therapy, stereotactic body radiation therapy, brachytherapy, metastasis direct therapy, hydrogel spacer

## Abstract

A recent approach to radiotherapy for prostate cancer is the administration of high doses of radiation to the prostate while minimizing the risk of side effects. Thus, image-guided radiotherapy utilizes advanced imaging techniques and is a feasible strategy for increasing the radiation dose. New radioactive particles are another approach to achieving high doses and safe procedures. Prostate brachytherapy is currently considered as a combination therapy. Spacers are useful to protect adjacent organs, specifically the rectum, from excessive radiation exposure.

## 1. Introduction

The current trend in radiotherapy for patients with localized prostate cancer is characterized by the implementation of high-dose irradiation using advanced techniques to enable more precise targeting of the tumor while minimizing radiation exposure to adjacent organs, including the rectum, bladder, and urethra [1]. Radiotherapy is a conventional treatment option for localized prostate cancer, and management approaches vary depending on the patient’s National Comprehensive Cancer Network (NCCN) risk [2,3].

According to previous trials, radical prostatectomy and external beam radiation therapy (EBRT) are similarly effective in terms of overall survival (OS), disease-specific survival (DSS), biochemical relapse-free survival (bRFS), and quality of life (QOL) for localized prostate cancer [4,5].

Radiotherapy may also be combined with other treatments, such as androgen deprivation therapy (ADT), to enhance survival outcomes, even in high-risk patients [6].

In the first section, we summarize the treatment options for radiotherapy for each risk factor for localized prostate cancer. Advanced irradiation techniques are described in the second section, and treatment-related adverse events (AEs) are described in the last section.

## 2. Radiotherapy for Each Risk of Localized Prostate Cancer

**Figure 1:**
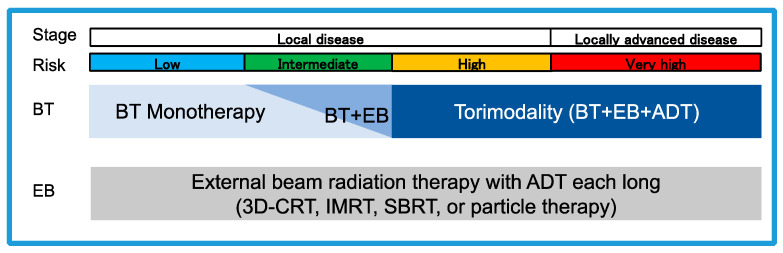
A perspective view of radiation therapy for patients with localized prostate cancer. BT, brachytherapy: EB, external beam radiation therapy; ADT, androgen deprivation therapy; 3D-CRT, three-dimensional conformal radiation therapy: IMRT, intensity modulated radiation therapy; SBRI, stereotache body radiation therapy.

Radiotherapy is the standard treatment for prostate cancer. Its treatment modality was applied to each risk (Figure 1).

### 2.1. Low-Risk Prostate Cancer

EBRT and low-dose rate (LDR) brachytherapy are the preferred primary treatments for prostate cancer [7,8,9]. EBRT has shown promising results, with a 10-year bRFS rate of approximately 85% in patients with low-risk prostate cancer [10]. Brachytherapy is also an effective treatment option with a 10-year bRFS rate of approximately 90% [11]. In selected cases, including elderly patients, stereotactic body radiation therapy (SBRT) was considered a viable treatment option [12].

### 2.2. Intermediate-Risk Prostate Cancer

The primary treatment approach for patients with intermediate risk depends on the patient’s condition and preferences and may include EBRT, brachytherapy, or a combination of both. EBRT can result in a 10-year bRFS rate of up to 75% [13], while brachytherapy can achieve a 10-year bRFS rate of up to 80% [14]. Combination therapies, such as EBRT and brachytherapy or EBRT and ADT, have shown better bRFS rates than monotherapy [15]. For instance, a 10-year bRFS rate of 62% was reported for the combination of EBRT and ADT, compared with 39% for EBRT alone [16]. Hypofractionated radiation therapy, which can irradiate higher doses of radiation in fewer treatment sessions, is an option for intermediate-risk patients [17]. SBRT could be an option for elderly patients [18].

### 2.3. High-Risk Prostate Cancer

Since EBRT has demonstrated a limited 10-year bRFS rate of 60% in patients with high-risk prostate cancer [19], EBRT combined with ADT is expected to improve biochemical control rates. The Radiation Therapy Oncology Group (RTOG) trial 92-02 revealed a 10-year bRFS rate of 74% with the combination of EBRT and ADT compared to 52% with EBRT alone [20]. Additionally, the Canadian Cancer Trials Group (CCTG) PR.3/MRC UK trial reported that a longer course of hormonal therapy improved cancer control and survival compared with a shorter course [7]. A combination treatment consisting of EBRT, brachytherapy, and ADT is also an option for better outcomes [21]. High-dose-rate (HDR) brachytherapy may effectively achieve biochemical control in high-risk prostate cancer, with a 10-year bRFS rate of up to 60% [22].

### 2.4. Very High-Risk Prostate Cancer

Radiotherapy is typically combined with ADT for patients with high-risk prostate cancer [23]. High-dose radiation therapy, including intensity-modulated radiotherapy (IMRT) and SBRT, effectively achieves biochemical control in very high-risk prostate cancer, with 5-year bRFS rates of up to 50% [24]. The trimodal combination of EBRT, ADT, and brachytherapy has been shown to improve the bRFS [25].

## 3. Technical Advancement of Radiotherapy

Since high-dose irradiation for prostate cancer has been proven to result in better treatment outcomes (Table 1), recent advancements in radiation techniques have been applied to patients with prostate cancer. IMRT or its additional technique of volumetric-modulated arc radiation therapy (VMAT) with image-guided radiation therapy (IGRT) is currently widely recognized as a reasonable treatment approach for EBRT [26].

### 3.1. IMRT

IMRT is a type of radiation therapy that utilizes computer-controlled radiation beams to irradiate a specific area with different radiation intensities for each specific area [27]. Radiation is delivered through multiple fixed-angle beams conforming to the prostate [28]. The intensity of each beam varies based on the specific targeted area. This approach enables precise tumor irradiation while minimizing exposure to the surrounding organs [29].

Several randomized studies have reported feasible outcomes (Table 2). The RTOG trial 0126, which compared conventional EBRT to IMRT in patients with localized prostate cancer [30], found that IMRT was associated with fewer AEs and improved QOL [31]. The NCT02257827 trial was a randomized controlled trial that compared IMRT to three-dimensional conformal radiation therapy (3DCRT) for patients with localized prostate cancer [32]. The primary endpoint was late toxicity, and the incidences of grade 2 or higher genitourinary (GU) and gastrointestinal (GI) toxicity at 6 months post-treatment were 3% and 1% in the IMRT group and 4% and 9% in the 3DCRT group, respectively. The 5-year bRFS rates did not differ between the IMRT and 3DCRT arms (95.4% and 94.3%, respectively).

Prostate cancer has high radiation-fraction sensitivity, which provides a therapeutic advantage for hypofractionated treatment. IMRT was suitable for the hypofractionated approach (Table 3). The Hypofractionated Versus Conventionally Fractionated Radiotherapy for Prostate Cancer trial was a randomized controlled trial comparing hypofractionated IMRT (H-IMRT) and conventional fractionated radiation therapy (CFRT) in 820 men with intermediate- to high-risk localized prostate cancer [33]. The results showed that H-IMRT was associated with a similar OS rate to CFRT at a median follow-up of 5 years, with similar rates of bRFS (80.5% vs. 77.1%) and an equivocal risk of late toxicity to CFRT [34]. H-IMRT was compared with CFRT in 3216 men with localized prostate cancer in the CHHiP trial [35]. H-IMRT was associated with similar bRFS rates as CFRT at a median follow-up of 62.4 months, with an estimated 5-year bRFS rate of 88.3% for H-IMRT and 90.6% for CFRT. The study also found that H-IMRT was not associated with an increased risk of toxicity compared with CFRT [36].

Several studies have shown that IMRT achieves favorable survival outcomes and can reduce the risk of AEs compared with conventional radiation therapy techniques [37] (Table 2 and Table 3).

### 3.2. IGRT

IGRT is a radiation treatment approach that utilizes images, such as computed tomography scans or magnetic resonance imaging, to guide the radiation delivery process [38]. This imaging modality allows for highly precise targeting of the tumor, which can result in improved treatment outcomes [39]. IGRT also helps minimize the risk of radiation exposure to healthy organs and can help minimize AEs such as urinary incontinence and bowel problems [40].

The effectiveness of IGRT in treating localized prostate cancer has been investigated in several randomized controlled trials (Table 4). A randomized safety trial conducted by the Honover group found that IGRT was much freer of acute GI symptoms (43% vs. 19%, *p* = 0.0012), although the grade 2 or higher GI toxicity rate did not differ [41].

IGRT can improve the accuracy and precision of radiation therapy for prostate cancer, leading to equivalent disease control and fewer AEs.

### 3.3. VMAT

VMAT is a type of IMRT that uses a rotating gantry to irradiate a continuous arc rather than delivering radiation from multiple fixed angles [42]. This technique allows for more precise irradiation while reducing treatment time. A linear accelerator rotates around the patient and irradiates from multiple angles while adjusting the radiation intensity to accurately target the tumor and minimize radiation exposure to surrounding healthy organs [43].

The use of fractionated radiation therapy for cancer treatment takes advantage of the differences in the DNA repair capacities of normal and tumor cells [44]. Slowly proliferating cells are sensitive to an increased dose per fraction, and a meta-analysis of 11 studies with over 8000 patients suggested that hypofractionated radiation therapy may be more effective for prostate cancer, which has a slower proliferation rate, than conventional fractions of 1.8–2 Gy [45] (Table 5). Additionally, hypofractionation is more convenient for patients and less costly [46].

Hypofractionated VMAT (H-VMAT) can reduce the overall treatment time and improve patient convenience [47]. H-VMAT has been shown to be an effective treatment for prostate cancer, with outcomes similar to those of conventional radiotherapy [48] (Table 5).

In summary, H-VMAT is expected to offer several potential benefits over conventional radiotherapy, such as shorter treatment duration, improved disease control, and reduced AEs.

### 3.4. SBRT

SBRT is focused radiotherapy that provides high doses of radiation to tumors in a small number of treatment sessions [49] (Table 6). SBRT typically involves a short treatment schedule of five or fewer sessions, whereas H-IMRT usually requires 15–20 treatment sessions [50].

A phase III randomized PACE-B trial comparing SBRT with conventional radiation therapy for patients with low- or intermediate-risk prostate cancer showed that the 2-year toxicity rates were similar for five fraction SBRT and conventional schedules [51] (Table 6). Concerning bRFS, 38 unique prospective series were identified, comprising 6116 patients [52]. The median follow-up duration was 39 months for all patients (range, 12–115 months). Overall, the 5- and 7-year bRFS rates were 95.3% (95% confidence interval [CI]: 91.3–97.5%) and 93.7% (95% CI: 91.4–95.5%), respectively.

SBRT delivers a higher radiation dose per treatment session and uses more precise targeting technology than H-VMAT, allowing for more accurate irradiation [53].

### 3.5. Brachytherapy

Brachytherapy involves the direct placement of tiny radioactive seeds into the prostate. These seeds emit radiation that induces apoptosis of cancer cells while minimizing radiation exposure to healthy organs besides the prostate [15]. Two types of brachytherapy, LDR and HDR, were administered to patients with prostate cancer [54] (Table 7).

#### 3.5.1. Permanent Brachytherapy

LDR brachytherapy involves permanently implanting tiny radioactive seeds (made of iodine-125 or palladium-103) that emit LDR brachytherapy over several months and gradually become inactive [55]. These seeds deliver a precise and targeted radiation dose to the prostate gland while sparing healthy tissues. This procedure is typically completed within a few hours.

LDR brachytherapy confers a favorable clinical outcome. As expected, 10-year bRFS rates of up to 98% and 90% were observed among patients presenting with low- and intermediate-risk prostate cancer, respectively [56,57] (Table 7). The RTOG 0232 trial compared brachytherapy and EBRT to brachytherapy alone in intermediate-risk prostate cancer patients and did not find improved biochemical progression-free survival [58] (Table 7).

LDR brachytherapy delivers the highest radiation dose directly to the prostate gland, minimizing exposure to nearby healthy tissues and reducing the risk of side effects, such as urinary and bowel dysfunction [59] (Table 7). In addition, it requires a shorter treatment time than EBRT, which improves patient convenience [60].

#### 3.5.2. HDR Brachytherapy

HDR brachytherapy involves temporarily inserting a small radioactive source into the prostate for a few minutes, emitting high doses of radiation to the target cancer cells [61]. This procedure requires anesthesia and several sessions over a few days.

In a meta-analysis of 2123 patients who underwent LDR brachytherapy, 40% were classified as low-risk, 40% as intermediate-risk, and 20% as high-risk patients based on NCCN [62]. The 5-year bRFS rate was 95%. After controlling for publication bias, an adjusted rate of 96% was achieved. The estimated adjusted rates of late grade 3 GU and GI toxicities were 2% and 0.3%, respectively.

Decreasing the frequency of treatment was considered even in LDR brachytherapy, and a randomized trial was undertaken to assess the frequency of HDR brachytherapy for intermediate-risk prostate cancer (Table 7). However, the findings suggested that a single fraction of HDR brachytherapy was inferior to two sessions regarding bRFS and toxicity rates [63].

#### 3.5.3. Trimodalilty Brachytherapy (Trimodality)

Trimodal therapy, which integrates EBRT, Brachytherapy, and ADT, is commonly used to treat patients with locally advanced prostate cancer who are not candidates for surgical intervention [64]. Although this approach may increase the effectiveness of cancer control compared with individual modalities, it may also increase the risk of adverse effects [65].

The Androgen Suppression Combined with Elective Nodal and Dose-Escalated Radiation Therapy trial compared brachytherapy with ADT to EBRT and a combination of both in terms of trimodality in patients with intermediate- or high-risk prostate cancer [66] (Table 7). Torimodality showed improved bRFS rates; however, higher rates of GU toxicity were also observed [67]. Another ongoing randomized study, the TRIP study from Japan, is expected to provide additional insight into the potency and limitations of adding 2 years of adjuvant hormone therapy to this trimodality approach and establish an appropriate treatment strategy for high-risk prostate cancer [68].

### 3.6. Particle Radiotherapy

Particle therapy, including proton and heavy-ion radiation therapies, is widely accepted as a feasible option for radiotherapy in patients with localized prostate cancer [69]. Proton beam therapy (PBT) is a form of radiotherapy that utilizes high-energy protons to target tumors as opposed to X-rays. Protons can be directed more precisely toward the tumor site and have a lower probability of damaging adjacent organs [70,71] (Table 8). Heavy ion radiotherapy is a specialized radiation therapy that utilizes high-energy ions such as carbon or helium [72]. This treatment involves directing a focused stream of charged particles to the tumor to deliver a potent radiation dose while preserving the surrounding organs [73].

J-CROS1501PR, a single-arm prospective study of carbon-ion radiation therapy (CIRT), showed that the 5-year bRFS rates were 92%, 89%, and 92% in low-, intermediate-, and high-risk patients, respectively. The incidence rates of grade 2 late GU and GI toxicities were 4.6% and 0.4%, respectively [74].

The meta-analysis included 33 studies involving 54,101 participants, with 13 studies focusing on CIRT and 20 on PBT [75]. This meta-analysis revealed high local control rates and bRFS rates for CIRT. However, the certainty of the evidence was very low. The authors concluded that while the available evidence suggests that CIRT and PBT may improve OS and local control rates and reduce toxicity compared to photon radiotherapy, more high-quality controlled studies are needed to provide confident evidence in the future [75].

## 4. Management for AEs Caused by Radiotherapy

Despite being a widely used and effective treatment option for prostate cancer, radiotherapy may cause side effects and complications [76]. Potential issues associated with radiation therapy for prostate cancer include the following.

### 4.1. Urinary Problems

Radiotherapy is a ubiquitous therapeutic modality for managing prostate cancer; however, its administration may be associated with undesirable urinary sequelae. The urinary tract encompasses the bladder, urethra, and kidneys, and ionizing radiation adversely impacts these organs [77,78]. Common urinary side effects of radiation therapy for prostate cancer include the following:

Urinary frequency: This annoying symptom denotes the need to void more frequently than is customary.

Urgency: This is the abrupt onset of an intense desire to urinate that is difficult to control.

Incontinence: This refers to the involuntary leakage of urine.

Dysuria: This symptom means painful or difficult urination.

Hematuria: This represents the presence of blood in the urine [78].

The severity of these urinary symptoms may vary depending on the radiation dose, location of the irradiated region within the urinary tract, and individual patient characteristics. These adverse effects may commence during therapy and persist for several weeks or months after the cessation [79].

To alleviate these complications, patients may need to implement various lifestyle changes such as augmenting fluid intake, abstaining from caffeine and alcohol consumption, and engaging in pelvic floor muscle exercises [80]. In addition, pharmacological interventions may be prescribed to alleviate GU toxicity.

### 4.2. Bowel Problems

Radiotherapy administered to treat prostate cancer may induce GI symptoms in some patients. Radiation can cause irritation and inflammation of the rectal mucosa, leading to a spectrum of symptoms commonly referred to as radiation proctitis [77]. The severity of GI symptoms may vary among patients, including but not limited to [81,82]:

Diarrhea: Patients may encounter frequent loose or watery bowel movements, which may be accompanied by the presence of blood.

Rectal pain: Patients may experience discomfort, pain, or pressure near the rectum during defecation.

Urgency and frequency: Patients may experience a sense of a pressing need to defecate and may need to do so more frequently than their typical routine.

Incontinence: Some patients may undergo a loss of voluntary control over bowel movements.

Straining: Patients may encounter difficulties evacuating their bowels or experience a sensation of bowel fullness [83].

Rectal bleeding: Radiation proctitis can trigger mild-to-severe bleeding from the rectal mucosa, ranging from mild to severe degrees [84].

Managing radiation-induced bowel symptoms requires a comprehensive approach that includes lifestyle modifications and medical interventions. Strategies to manage these symptoms are as follows [85]:

Increased intake of fruits, vegetables, whole grains, and legumes promotes bowel regularity as a dietary modification. Drink plenty of fluid to maintain adequate hydration and soften stool. Identify and avoid foods that worsen bowel symptoms, such as spicy or greasy foods, caffeine, alcohol, and foods with high fat content. As a medication, consider using stool softeners, such as docusate sodium, to alleviate constipation. Fiber supplements or mild laxatives such as psyllium may also be helpful.

### 4.3. Erectile Dysfunction

Ionizing radiation can injure surrounding anatomical structures, such as nerves and blood vessels, which are critical for attaining and maintaining erectile function [86]. This can lead to sexual dysfunction ranging from moderate difficulty with erection to complete impotence [87].

The extent of erectile dysfunction is multifactorial, and factors such as the patient’s age, general health status, ADT, and the type and magnitude of the administered radiation play a role [88]. Radiotherapy has acute and long-term effects on sexual function. The acute effects include reduced libido, challenges in achieving and sustaining erections, and a decrease in the quality of erections [89]. In contrast, long-term effects can be irreversible, manifesting as persistent erectile dysfunction and decreased overall sexual gratification.

Multiple treatment options exist for managing secondary erectile dysfunction from radiotherapy, including oral phosphodiesterase type 5 inhibitors, vacuum erection devices, intracavernosal injections, and penile prostheses [90,91]. Although these modalities can be efficacious in many men, they may also have potential adverse effects and may not be suitable for all patients. Hence, it is essential that patients have a detailed discussion with a urologist to determine the most appropriate therapeutic approach to meet their individual needs [92].

### 4.4. Fatigue

Radiotherapy has the potential to elicit fatigue, which can range from mild to severe and persist for several weeks or even months [93]. Fatigue in patients with prostate cancer undergoing radiation therapy may arise owing to various mechanisms. One mechanism involves the impairment of healthy organs. Radiotherapy eradicates cancerous cells while adversely affecting normal cells in the surrounding area, including cells in the bone marrow responsible for producing red blood cells [94]. This depletion of red blood cells can lead to anemia, a condition characterized by inadequate oxygen-carrying red blood cells, ultimately resulting in fatigue [95].

Radiotherapy can also initiate inflammation in the body, which contributes to fatigue. The release of pro-inflammatory cytokines in response to radiation can trigger an inflammatory response [96]. Inflammation can activate the immune system, releasing chemicals that induce fatigue [97].

Disturbance of the body’s circadian rhythm is another factor that can contribute to radiation-induced fatigue. Radiation therapy disrupts the normal sleep–wake cycle, which can lead to fatigue and other sleep-related disturbances [98].

It is advisable to rest when feeling tired to manage radiation-induced fatigue because physical activity can exacerbate fatigue [99]. Short naps during the day and sufficient nighttime sleep are also beneficial. Exercise, specifically light to moderate exercise such as walking or yoga, can help mitigate fatigue [100]. However, before starting an exercise regimen, it is important to consult a doctor [101].

Maintaining a balanced and nutritious diet can help manage fatigue [102]. Foods high in protein, fiber, and complex carbohydrates are beneficial for maintaining energy levels. However, foods high in sugar and caffeine can cause energy crashes and should be avoided.

In some cases, medications may be prescribed to help manage fatigue. Stimulants such as modafinil or methylphenidate are recommended to boost energy levels [103]. Psychological support can be beneficial because fatigue can negatively impact mental health. Support groups, counseling, and therapy can help mitigate stress and anxiety, which can contribute to fatigue [104].

Energy conservation is another strategy to manage fatigue. Prioritizing tasks, delegating responsibilities when possible, and taking breaks as needed are all effective methods for conserving energy [105].

### 4.5. Secondary Cancers

Although rare, radiotherapy can increase the risk of secondary cancers in treated areas or other body parts [106,107]. The risk of developing secondary cancer due to radiation therapy is relatively low and depends on various factors, such as the patient’s age, dose of radiation received, and location of radiation treatment [106]. The risk may also depend on whether the patient has received any prior cancer treatment, such as chemotherapy or surgery. It is important to note that while there is a risk of developing secondary cancer, the benefits of radiotherapy in treating prostate cancer typically outweigh the potential risks [108,109,110]. Moreover, a recent population-based cohort study suggested that IMRT for prostate cancer was not associated with an increased risk of second primary cancers [111].

### 4.6. Spacer

Space can mitigate the risk of rectal damage during radiotherapy for prostate cancer. Radiation therapy can affect adjacent healthy tissues and cause side effects such as rectal bleeding, diarrhea, and pain during bowel movements.

SpaceOAR^®^ is an injected hydrogel, a polyethylene glycol, that creates a space between the prostate gland and the rectum, minimizing the amount of radiation delivered to the rectum [112]. This unique material reduces the risk of rectal damage and improves the safety and effectiveness of radiotherapy. Clinical studies have shown SpaceOAR^®^ to be an effective tool for reducing the risk of side effects associated with radiation therapy in prostate cancer [113]. It has also been used as a perirectal spacer [114]. However, like any treatment, space has potential risks and complications, and patients should discuss its use with qualified medical professionals [112].

## 5. Conclusions

Radiotherapy is a conventional treatment for localized prostate cancer. The selection of a specific type of radiation therapy depends on the stage and risk category of cancer and the patient’s unique medical and social profile. Radiotherapy is generally a beneficial alternative treatment for prostate cancer, and advancements in technology and methods are constantly enhancing results and reducing side effects.

## Figures and Tables

**Table 1:** Randomized trials evaluating external beam radiation therapy dose escalation for localized prostate cancer.

Study	Year	Patient’s Number	PCa Characteristics	Dose (Gy)	ADT	bRFS (Phoenix)		Toxicity			
PROG/ACR 95-09	2010	196 vs. 197	low (58%), intermediate (37%), and high (4%) risk	79.2 vs. 70.2	-	10-year	82.6% vs. 68.0%	6-month grade ≥ 2 GU toxicity	29% vs. 25%	6-month grade ≥ 2 GI toxicity	24% vs. 13%
GETUG 06	2011	153 vs. 153	intermediate (28.9%), and high (71.1%) risk	80 vs. 70	-	5-year	72% vs. 61%	grade ≥ 2 GU toxicity	17.5% vs. 10%	grade ≥ 2 GI toxicity	19.5% vs. 14%
MRC RT01	2014	422 vs. 421	low (19%), intermediate (37%), and high (43%) risk	74 vs. 64	physician decision	10-year	55% vs. 43%				
Dutch CKVO96-10	2014	333 vs. 331	low (17.9%), intermediate (27.0%), and high (55.1%) risk	78 vs. 68	-	10-year	49% vs. 43%				
RTOG 0126	2018	748 vs. 751	low or intermediate risk	79.2 vs. 70.2	-	8-year	80% vs. 75%	5-year grade ≥ 2 GU toxicity	12% vs. 7%	5-year grade ≥ 2 GI toxicity	21% vs. 15%
MD Anderson study	2019	151 vs. 150	low (20.6%), intermediate (45.8%), and high (33.6%) risk	78 vs. 70	-	15-year	92.9% vs. 87.7%				
FLAME Trial	2021	284 vs. 287	low (1.1%), intermediate (15.1%), and high (83.9%) risk	77 + focal boost vs. 77	physician decision	5-year	92% vs. 85%	late grade ≥ 2 GU toxicity	27.8% vs. 23.0%	late grade ≥ 2 GI toxicity	12.7% vs. 12.2%

PCa, prostate cancer; Gy, gray; ADT, androgen deprivation therapy; bRFS, biochemical relapse-free survival; GU, genitourinary; GI, gastrointestinal.

**Table 2:** Randomized trials evaluating intention-modulated radiation therapy for localized prostate cancer.

Study	Year	Patient’s Number	PCa Characteristics	Dose (Gy)	ADT	bRFS (Phoenix)		Toxicity			
NCT02257827	2016	109 vs. 106	low (43.7%), intermediate (21.9%), and high (34.4%) risk	70 (IMRT) vs. 70 (3DCRT)	2 years in intermediate- and high-risk patients	5-year	95.4% vs. 94.3%	6-month grade ≥ 2 GU toxicity	3% vs. 4%	6-month grade ≥ 2 GI toxicity	1% vs. 9%
The PROFIT trial	2017	608 vs. 598	intermediate risk	60 (IMRT) vs. 78 (3DCRT)	only 6% of all patients	5-year	85% vs. 85%	6-month grade ≥ 3 GU toxicity	2.1% vs. 3.0%	grade ≥ 3 GI toxicity	1.5% vs. 2.7%
RTOG 0126	2018	748 vs. 751	low or intermediate risk	79.2 vs. 70.2	-	8-year	80% vs. 75%	5-year grade ≥ 2 GU toxicity	12% vs. 7%	5-year grade ≥ 2 GI toxicity	21% vs. 15%
POP-RT	2021	110 vs. 114	high risk	68 + 50 vs. 68	2 years	5 year	95.0% vs. 81.2%	late grade ≥ 2 GU toxicity	20.0% vs. 9.0%	late grade ≥ 2 GI toxicity	8.2% vs. 4.5%

PCa, prostate cancer; Gy, gray; ADT, androgen deprivation therapy; bRFS, biochemical relapse-free survival; IMRT, intensity modulated radiation therapy; 3DCRT, three-dimensional conformal radiation therapy; GU, genitourinary; GI, gastrointestinal.

**Table 3:** Randomized trials evaluating hypofractionated intensity-modulated radiation therapy for localized prostate cancer.

Study	Year	Patient’s Number	PCa Characteristics	Dose (Gy)	ADT	bRFS (Phoenix)		Toxicity			
HYPRO trial	2016	407 vs. 397	intermediate (26.2%) and high (73.8%) risk	64.6 in 19 f vs. 78.0 in 39 f	each institutional protocol	5-year	80.5% vs. 77.1%				
CHHiP trial	2016	1074 and 1077 vs. 1065	low (15.0%), intermediate (73.0%), and high (12.0%) risk	60 in 20 f or 57 in 19 f vs. 74 in 37 f	3–6 months	5-year	90.6%, 85.9% vs. 88.3%	2-year grade ≥ 2 GU toxicity	2%, 1% vs. 1%	2-year grade ≥ 2 GI toxicity	3%, 2% vs. 4%

PCa, prostate cancer; Gy, gray; ADT, androgen deprivation therapy; bRFS, biochemical relapse-free survival; f, fraction; GU, genitourinary; GI, gastrointestinal.

**Table 4:** Randomized trials evaluating image guided radiation therapy for localized prostate cancer.

Study	Year	Patient’s Number	PCa Characteristics	Treatment Methods	ADT	bRFS (Phoenix)		Toxicity			
Hannover study	2016	102 vs. 96	low (15.2%), intermediate (34.3%), and high (50.5%) risk	IGRT vs. non-IGRT	physician decision			late grade ≥ 2 GU toxicity	34% vs. 34%	late grade ≥ 2 GI toxicity	19% vs. 31%
RIC-trial	2018	125 vs. 125	intermediate (39.2%), and high (60.8%) risk	IGRT daily vs. IGRT weekly	6 months in intermediate- and 3 years in high-risk		89.3% vs. 84.6%				
STIC-IGRT trial	2018	234 vs. 236	low (0.6%), intermediate (69.1%), and high (32.0%) risk	IGRT daily vs. IGRT weekly	physician decision	5-year	91% vs. 79%	5-year grade ≥ 2 GU toxicity	14% vs. 18%	5-year grade ≥ 2 GI toxicity	10% vs. 13%
CHHiP	2020	137 and 108 vs. 48	low (11.9%), intermediate (77.5%), and high (10.6%) risk	IGRT-S and -R vs. non-IGRT	3–6 months			2-year grade ≥ 2 GU toxicity	4.6%, 3.9% vs. 8.4%	2-year grade ≥ 2 GI toxicity	8.3%, 5.8% vs. 8.3%

PCa, prostate cancer; Gy, gray; ADT, androgen deprivation therapy; bRFS, biochemical relapse-free survival; IGRT, image-guided radiation therapy; IGRT-S, standard image-guided radiation therapy; IGRT-R, reduced image-guided radiation therapy; GU, genitourinary; GI, gastrointestinal.

**Table 5:** Randomized trials evaluating hypofractionated and dose-escalated intensity-modulated radiation therapy for localized prostate cancer.

Study	Year	Patient’s Number	PCa Characteristics	Dose (Gy)	ADT	bRFS (Phoenix)		Toxicity			
Marilia Medical School	2016	109 vs. 106	low (43.7%), intermediate (21.9%) and high (34.4%)	70 (IMRT) vs. 70 (3DCRT)	6 months in intermediate- and 2 years in high-risk	5-year	95.4% vs. 94.3%	6-month grade ≥ 2 GI toxicity	1% vs. 9%	6-month grade ≥ 2 GU toxicity	3% vs. 4%
HYPRO trial	2016	407 vs. 397	intermediate (26.2%) and high (73.8%)	64.6 in 19 f (VMAT) vs. 78.0 in 39 f	each institutional protocol	5-year	80.5% vs. 77.1%				
CHHiP trial	2016	1074 and 1077 vs. 1065	low (15.0%), intermediate (73.0%), and high (12.0%) risk	60 in 20 f or 57 in 19 f vs. 74 in 37 f	3–6 months	5-year	90.6%, 85.9% vs. 88.3%	2-year grade ≥ 2 GI toxicity	3%, 2% vs. 4%	2-year grade ≥ 2 GU toxicity	2%, 1% vs. 1%
MD Anderson study	2018	103 vs. 103	low (25.7%), intermediate (66.2%), and high (0.9%) risk	72 in 30 f vs. 75.6 in 42 f	for patients with PSA levels > 10 ng/mL or cT3 disease	8-year	89.3% vs. 84.6%	8-year grade ≥ 2 GI toxicity	12.6% vs. 5.0%	8-year grade ≥ 2 GU toxicity	15.1% vs. 16.4%
NCT00062309	2020	151 vs. 152	low (9.2%), intermediate (62.4%) and high (28.4%)	70.2 in 26 f vs. 76 in 38 f	4 months in intermediate- and 2 years in high-risk	10-year	74.6% vs. 78.9%				

PCa, prostate cancer; Gy, gray; ADT, androgen deprivation therapy; bRFS, biochemical relapse-free survival; IMRT, intensity modulated radiation therapy; 3DCRT, three-dimensional conformal radiation therapy; f, fraction; VMAT, volumetric modulated arc therapy; GU, genitourinary; GI, gastrointestinal; PSA, prostate specific antigen; T, tumor.

**Table 6:** Randomized trials evaluating stereotactic body radiation therapy for localized prostate cancer.

Study	Year	Patient’s Number	PCa Characteristics	Dose (Gy)	ADT	bRFS (Phoenix)		Toxicity			
HYPO-RT-PC	2019	589 vs. 591	intermediate (89%) and high (11%) risk	42.7 in 7 f vs. 78.0 in 39 f	-	5 year	84% vs. 84%	2-year grade ≥ 2 GU toxicity	13% vs. 9%	2-year grade ≥ 2 GI toxicity	6% vs. 5%
PACE-B	2022	416 vs. 433	low (8.0%) and intermediate (92.0%) risk	36.25 in 5 f vs. 78 in 39 f or 62 in 20 f	-			2-year grade ≥ 2 GU toxicity	3% vs. 2%	2-year grade ≥ 2 GI toxicity	2% vs. 3%

PCa, prostate cancer; Gy, gray; ADT, androgen deprivation therapy; bRFS, biochemical relapse-free survival; f, fraction; GU, genitourinary; GI, gastrointestinal.

**Table 7:** Randomized trials evaluating brachytherapy for localized prostate cancer.

Study	Year	Treatment	Patient’s Number	PCa Characteristics	Treatment Methods	ADT	bRFS (Phoenix)		Toxicity			
San Paolo Hospital	2009	LDR	85 vs. 89		BT vs. RP	-	5-year	91.0% vs. 91.7%				
RTOG 0232	2016	LDR	287 vs. 292	Intermediate risk	EBRT + BT vs. BT	?	5-year	85% vs. 86% (PFS)				
ISRCTN98241100	2012	HDR	110 vs. 106	low (4.2%), intermediate (42.1%) and high (53.7%)	EBRT + HDR-BT vs. EBRT	6 months in low/intermediate risk and up to 3 years in high-risk	12-year	69% vs. 49%	6-year grade ≥ 3 GU toxicity	11% vs. 4%	6-year grade ≥ 3 GI toxicity	0.9% vs. 0.8%
NCT01890096	2020	HDR	87 vs. 83	low (19.4%) and intermediate (80.6%) risk	19 Gy in 1 f vs. 27 Gy in 2 f	physician decision	5-year	73.5% vs. 95%	late grade ≥ 2 GU toxicity	45% vs. 45%		
ASCENDE-RT	2017	Torimodality	198 vs. 200	intermediate (30.7%) and high (69.3%) risk	EBRT + LDR-BT vs. DE-EBRT	12 months	7-year	85% vs. 76%	late grade ≥ 2 GU toxicity	32.8% vs. 20.6%	late grade ≥ 2 GI toxicity	31.3% vs. 20.2%

PCa, prostate cancer; Gy, gray; ADT, androgen deprivation therapy; bRFS, biochemical relapse-free survival; LDR, low dose rate; HDR, high dose rate; BT, brachytherapy; RP, radical prostatectomy; EBRT, external beam radiation therapy; f, fraction; DE-EBRT, dose-escalated external beam radiation therapy; GU, genitourinary; GI, gastrointestinal.

**Table 8:** Randomized trials evaluating particle therapy for localized prostate cancer.

Study	Year	Treatment	Patient’s Number	PCa Characteristics	Treatment Methods	ADT	bRFS (Phoenix)		Toxicity			
IPI	2016	Proton and Carbon ion	46 vs. 46	low (23.1%), intermediate (59.3%) and high (17.6%)	Proton vs. Carbon ion	physician decision	8-year	50% vs. 26%	late grade ≥ 2 GU toxicity	21.7% vs. 13.3%	late grade ≥ 2 GI toxicity	8.7% vs. 2.2%

PCa, prostate cancer; Gy, gray; ADT, androgen deprivation therapy; bRFS, biochemical relapse-free survival; GU, genitourinary; GI, gastrointestinal.
